# Extract from Notes of Obstetric Practice in the Chicago Hospital for Women and Children Since Its Opening, May 8th, 1865, to May 8th, 1877

**Published:** 1877-06

**Authors:** 


### *Extract from Notes of Obstetric Practice in the Chicago Hospital for women and Children since its Opening, May 8th*, 1865, *to May 8th*, 1877.

In charge of Mary H. Thompson, M. D.

The notes of 631 obstetric cases are obtained from what is left of the records after the "Great Fire," in 1871, and of those who have been treated since.

Of this number, 378 mothers are noted as multiparæ, and 116 primiparæ; the condition of the remainder is not recorded. The presentations are recorded in 425 cases; *two* were hand and foot; *two* were head and cord; *one* cord and foot; *two* were cord. *Six* were face; *one* breech and hand. *Six* were breech; *four* were foot. *One* was knee; *one* was placenta; *thirty-nine* vertex, and the remainder were simply noted as *head* presentations.

Accidents to women were as follows: *Two* died of septicæmia. *One*, pulmonary consumption. *One*, from a general cellulitis. *One*, from puerperal cellulitis and metritis. *One*, from puerperal metritis. *One*, from shock following turning after woman had been in labor eighteen hours, and membranes ruptured twelve hours. Cord presentation.

There were several cases of septicæmia and recovery which were not recorded.

There were *six* cases of puerperal convulsions. Two died; four recovered. Of those who recovered, two were bled and given chloroform; two took chloroform without venesection. Chloroform was administered to the two patients who died.

*Adherent placenta* occurred four times. *Severe hemorrhage* occurred *five times*, but never proved fatal. *Prolapsus of bladder* occurred once. *Thrombus of vagina*, as large as fœtal head at term, with spontaneous rupture occurred once. Clots were turned out and the part was compressed by tampon in vagina, and cavity afterward kept disinfected with injections containing carbolic acid. A *tumor* in anterior wall of vagina complicated one labor.

Cystitis came on after labor in one case. *Laceration* of the *cervix uteri* occurred in *two* cases. *Laceration* of the *perineum* complicated *fifteen* cases.

Accidents and injuries to children were as follows: *Fifteen* children were dead previous to labor; *four* died from difficult labor; *two* died of tetanus; *one* of marasmus; *one* from premature birth at six months; *one* from hemorrhage at the base of the umbilical cord; *three* from asphyxia; *two* were accidental.

One child, born at the fifth month, lived over one hour. There were two pairs of twins. One child had an hæmatoma.

The placenta was weighed in a large number of full term cases. The greatest weight was 3 pounds, and the least 3 ounces. The umbilical cord was measured, and the greatest length was four and a half feet; the least, four inches.

Two *knots* were found in one long cord.

It is a rule in this hospital, that the urine of every pregnant woman, who comes in previous to labor, shall be examined for albumen. While it was done in a large majority of cases, it was found but a very few times—not exceeding twelve. It always disappeared with the use of dry cups over the kidneys, repeated every fourth or fifth day, and the administration of the tincture of iron, in ten to fifteen drop doses, and if needed, some saline cathartic.

It was found in large quantities in *three* of the cases of convulsions, but these had been admitted during labor, or but a few hours previously.

